# How accurate are gender detection tools in predicting the gender for Chinese names? A study with 20,000 given names in Pinyin format

**DOI:** 10.5195/jmla.2022.1289

**Published:** 2022-04-01

**Authors:** Paul Sebo

**Affiliations:** 1 paulsebo@hotmail.com, Primary Care Unit, Faculty of Medicine, University of Geneva, Geneva, Switzerland

**Keywords:** accuracy, Chinese, gender detection, misclassification, name, name-to-gender, performance

## Abstract

**Objective:**

We recently showed that the gender detection tools NamSor, Gender API, and Wiki-Gendersort accurately predicted the gender of individuals with Western given names. Here, we aimed to evaluate the performance of these tools with Chinese given names in Pinyin format.

**Methods:**

We constructed two datasets for the purpose of the study. File #1 was created by randomly drawing 20,000 names from a gender-labeled database of 52,414 Chinese given names in Pinyin format. File #2, which contained 9,077 names, was created by removing from File #1 all unisex names that we were able to identify (i.e., those that were listed in the database as both male and female names). We recorded for both files the number of correct classifications (correct gender assigned to a name), misclassifications (wrong gender assigned to a name), and nonclassifications (no gender assigned). We then calculated the proportion of misclassifications and nonclassifications (errorCoded).

**Results:**

For File #1, errorCoded was 53% for NamSor, 65% for Gender API, and 90% for Wiki-Gendersort. For File #2, errorCoded was 43% for NamSor, 66% for Gender API, and 94% for Wiki-Gendersort.

**Conclusion:**

We found that all three gender detection tools inaccurately predicted the gender of individuals with Chinese given names in Pinyin format and therefore should not be used in this population.

## INTRODUCTION

Nowadays, many researchers use gender detection tools for their studies, as the tools give the advantage of saving researchers time and resources when they need to integrate the gender variable into their databases. These tools are particularly useful for exploring gender inequalities in research. For example, a study using genderize.io showed that only 16% of editorial board members of emergency medicine journals were women [1]. A second study using Gender API found that the proportion of women as first authors of scientific articles published between 2016 and 2020 was higher in primary health care journals than in general internal medicine journals (54% versus 41%) [2].

If accurate, these tools could better combat gender inequalities in the academic world by expanding knowledge about the inequalities. Although the medical profession has increased the proportion of women in recent decades [3], female physicians continue to face barriers throughout their careers that are difficult to overcome, for example, funding for their research projects [4] or career progression [5].

Different gender detection tools have several features in common. They are relatively easy to use for interested researchers or librarians, as they do not require extensive computer knowledge. In addition, they are fast and can be applied to large datasets. Despite their advantages, their algorithms are often complex and difficult for nonspecialists to understand. In general, they all rely on large, often open-access name repositories and attempt to refine the results obtained by using additional information about the cultural context, mainly family name or country of origin [6]. For example, the first name Andrea is predominantly female in many countries but not in Italy.

Until recently, there were little data in the literature regarding the performance of gender detection tools [6]. In a previous paper, we compared tools that allowed large amounts of data to be uploaded as Excel, CSV, or text files [7]. Three of these tools showed high performance in gender prediction: Gender API (https://gender-api.com/en/), NamSor (https://www.namsor.com/), and Wiki-Gendersort (https://github.com/nicolasberube/Wiki-Gendersort). The fourth tool evaluated in the study (genderize.io) proved to be unreliable. The accuracy of prediction may depend on the origin of the given names in the databases. As our previous study was based mainly on names from Western countries, detailed analysis of tool performance according to the origin of non-Western names was not feasible.

The lack of data on the performance of gender detection tools for Chinese names, in particular, is problematic. Indeed, the share of Chinese names in the queries performed by researchers with these tools is expected to grow significantly in the coming years given the vitality of research in China. China has become a scientific superpower due to massive public funding of research and a large number of researchers in science and technology [8]. It is now the second-largest funder of research and development after the United States [9]. In addition, about eight million students graduate from Chinese universities every year, and this number is expected to grow by 300% by 2030 [10]. Overall, China currently has more than two million scientists and engineers, and the number of publications by Chinese authors increased from 30,780 in 1996 to 684,048 in 2019 [11]. As such, it became the most scientifically productive country in the world, just ahead of the US. In 2019, China also ranked second in the world in terms of the number of high-quality research papers [12].

In view of the increasing share of Chinese researchers in scientific production, we aimed to evaluate whether the performance of Gender API, NamSor, and Wiki-Gendersort remains high when used with Chinese given names.

## METHODS

### Study population

The study relied on a gender-labeled database of 172,624 Chinese given names in Pinyin format [13]. Pinyin is the official and internationally accepted phonetic representation of Chinese words [14, 15]. It converts Chinese characters into Latin letters using a transcription method called romanization. Unfortunately, the romanization of Chinese names leads to a loss of information; two different given names in Chinese characters can be written in the same way in Pinyin. However, we used the Pinyin format in this study because it is generally the standard used in the academic world, for example to list the authors of scientific articles.

The gender-labeled database was originally compiled from several public sources in China. The creator of the database did not provide detailed information about the public sources used and the procedure followed to create the database. Pypinyin, a Python module that translates Chinese characters into Pinyin format, was used to complete the file with Pinyin given names. The Pypinyin module is available for free on GitHub [16].

After removing all duplicates except for the first occurrence, there were 52,414 given names remaining in the database, from which we drew 20,000 names at random to create the main file we used in the study (File #1). Randomization was done in Excel using the random number generator, and the randomly drawn names were transferred to Stata to perform the analyses. Unisex names were kept in two copies (one associated with the female gender, the other with the male gender). The design of the study did not allow us to identify unisex names that were listed in the database only as female or male. The database contained 19,768 names of two Chinese characters (98.8%) and 232 names of one Chinese character (1.2%). We created a second file (File #2) containing 9,077 names by removing from File #1 all unisex names that we were able to identify (i.e., those that were listed in the database as both male and female names).

### Gender detection tools

To be included in the study, gender detection tools had to accept name files (in Excel, CSV, or text format) and be at least partially free of charge (i.e., free up to a certain number of queries per day, week, or month). The free and paid versions of the tools are identical; the only difference is the number of queries allowed. Only tools that were shown to be accurate in our previous study (with mostly Western names) were included in the current study [7]. In the absence of recognized criteria, we defined a tool as accurate if the proportion of both misclassifications (i.e., wrong gender assigned to a given name) and nonclassifications (i.e., unrecognized given names) was <10%.

Three tools fulfilled these conditions: Gender API (free up to 500 queries per month, inaccuracy rate 1.8%) [17], NamSor (free up to 5,000 queries per month, inaccuracy rate 2.0%) [18], and Wiki-Gendersort (completely free, inaccuracy rate 6.6%) [19]. For the three tools selected, the query response options were female, male, or unknown (gender could not be determined).

Unlike Wiki-Gendersort, Gender API and NamSor provide additional parameters for gender estimation. For Gender API, these parameters are “samples,” which give the number of database records that match the query, and “accuracy,” which determines the precision of the estimate. For NamSor, there are three additional parameters: “score,” “probabilityCalibrated,” and “genderScale.” The first parameter (“score”) is based on the relative probability between the predicted value and the second-best alternative and is calculated as log [probability (best) / probability (second_best)]. This score is then normalized to a [0–1] range, which can be interpreted directly as a probability (“probabilityCalibrated”), with a probability of 1 corresponding to perfect precision. The third parameter (“genderScale”) evaluates the probability that a name is male or female (i.e., the higher the probability that the name is male or female, the closer this parameter is to -1 or 1, respectively). We did not use these additional parameters because, as discussed below, they did not modify the results of the study.

The three gender detection tools evaluated have the advantage that they can be used even by researchers and librarians with little computer knowledge. For example, Gender API [17] only requires the uploading of a database in Excel or CSV format (https://gender-api.com/en/excel-and-csv). After processing, a gender column is added to the initial file.

Until recently, NamSor [18] could not be easily used because it required the use of a connector (NamSor Custom Connector) via the free application Power BI Desktop. However, the tool now allows the uploading of a database in CSV or TXT format (https://v2.namsor.com/NamSorAPIv2/apiprocessor.html). For our study, we used the version with Power BI Desktop.

Finally, Wiki-Gendersort [19] requires the installation of the module on the computer and then the use of a specific function (file_assign) to assign a gender to a list of names in TXT format. We used Spyder, an open-source integrated development environment for programming in Python. In Spyder, once the module is installed on the computer, one must type the following code in the console to get a new TXT file (“first_names_output”) with the estimated gender for each name in the original file called “first_names”:

from Wiki_Gendersort import wiki_gendersort
WG = wiki_gendersort()
WG.file_assign('first_names.txt')

### Performance metrics

We estimated the accuracy of gender prediction by calculating four performance metrics that were based on the number of given names for which the gender was correctly assigned (correct classifications), the number of given names with misclassifications, and the number of given names without assignments (nonclassifications). These performance metrics were introduced by Wais and Santamaría & Mihaljević [6, 20]. To facilitate the calculation of the metrics, we constructed a confusion matrix, which is a table that describes the performance of a classification model on a set of test data for which the true values are known. In this table, we showed six different combinations of predicted and actual values: ff (actual gender: female, predicted gender: female) and mm (actual gender: male, predicted gender: male) corresponding to correct classifications, mf (actual gender: male, predicted gender: female) and fm (actual gender: female, predicted gender: male) corresponding to misclassifications, and fu (actual gender: female, predicted gender: unknown) and mu (actual gender: male, predicted gender: unknown) corresponding to nonclassifications (Table 1).

**Table 1:** Confusion matrix showing six possible classification outcomes

	Female (predicted)	Male (predicted)	Unknown (predicted)
Female (actual)	ff	fm	fu
Male (actual)	mf	mm	mu

The first metric, errorCoded ((fm + mf + mu + fu) / (mm + fm + mf + ff + mu + fu)), measures the proportion of both misclassifications and nonclassifications (i.e., the overall performance of the tool). The second metric, naCoded, ((mu + fu) / (mm + fm + mf + ff + mu + fu)), measures the proportion of nonclassifications. The third metric, errorCodedWithoutNA ((fm + mf) / (mm + fm + mf + ff)), measures the proportion of misclassifications after removing the nonclassifications. The fourth metric, errorGenderBias ((mf – fm) / (mm + fm + mf + ff)), estimates the direction of bias in gender prediction and is used to assess whether errors are more frequent with male or female names. A positive value implies that there are more male names misclassified as female than female names misclassified as male. There is no threshold above which this bias can be described as problematic.

For given names of two Chinese characters, we first used the full given name in Pinyin. Then, for each tool tested, we repeated the analyses with only the second Chinese character if the gender could not be determined with the full given name, as the second character more easily allows the determination of gender.

### Ethical considerations

As this study did not involve the collection of personal health-related data, it did not require ethical review according to current Swiss law.

## RESULTS

Tables 2 and 3 show the confusion matrices and performance metrics, respectively, of the three gender detection tools for File #1. The same data are shown in Tables 4 and 5 for File #2 (i.e., after removing all unisex given names that we were able to identify).

**Table 2:** Confusion matrices for gender detection tools (n=20,000 given names)

Gender detection tool	Classified as women n (%)	Classified as men n (%)	Not classified n (%)
Gender API			
Women	1,836 (22.8)	3,066 (38.1)	3,142 (39.1)
Men	1,974 (16.5)	5,173 (43.3)	4,809 (40.2)
NamSor			
Women	1,545 (19.2)	4,869 (60.5)	1,630 (20.3)
Men	1,635 (13.7)	7,950 (66.5)	2,371 (19.8)
Wiki-Gendersort			
Women	806 (10.0)	771 (9.6)	6,467 (80.4)
Men	941 (7.9)	1,140 (9.5)	9,875 (82.6)

**Table 3:** Performance metrics for gender detection tools (n=20,000 given names)

Gender detection tool	errorCoded	errorCodedWithoutNA	naCoded	errorGenderBias
Gender API	0.6496	0.4183	0.3976	−0.0906
NamSor	0.5253	0.4065	0.2001	−0.2021
Wiki-Gendersort	0.9027	0.4680	0.8171	0.0465

**Table 4:** Confusion matrices for gender detection tools after removing all unisex names we were able to identify (n=9,077 given names)

Gender detection tool	Classified as women n (%)	Classified as men n (%)	Not classified n (%)
Gender API			
Women	347 (13.8)	544 (21.7)	1,616 (64.5)
Men	472 (7.2)	2,777 (42.3)	3,321 (50.5)
NamSor			
Women	409 (16.3)	1,585 (63.2)	513 (20.5)
Men	501 (7.6)	4,791 (72.9)	1,278 (19.5)
Wiki-Gendersort			
Women	95 (3.8)	80 (3.2)	2,332 (93.0)
Men	244 (3.7)	451 (6.9)	5,875 (89.4)

**Table 5:** Performance metrics for gender detection tools after removing all unisex names we were able to identify (n=9,077 given names)

Gender detection tool	errorCoded	errorCodedWithoutNA	naCoded	errorGenderBias
Gender API	0.6558	0.2454	0.5439	−0.0174
NamSor	0.4271	0.2863	0.1973	−0.1488
Wiki-Gendersort	0.9400	0.3724	0.9042	0.1885

For File #1, the numbers of misclassifications and nonclassifications were high for both Gender API (n=5,040 and 7,951, respectively) and NamSor (n=6,504 and 4,001) (Table 2). For Wiki-Gendersort, the number of misclassifications was lower (n=1,712) but only because the number of nonclassifications was very high (n=16,342). For Gender API and Wiki-Gendersort, the proportion of nonclassifications was higher and the proportion of misclassifications lower for File #2 than for File #1, while for NamSor, the proportion of nonclassifications was similar and the proportion of misclassifications was lower for File #2 than for File #1.

The metrics in Table 2 confirmed the low performance of all three tools. Using errorCoded, which takes into account both types of inaccuracy (misclassifications and nonclassifications), the proportion of errors was 53% for NamSor, 65% for Gender API, and 90% for Wiki-Gendersort. The proportion of misclassifications was similar for the three tools (ranging from 41% to 47%), while the proportion of nonclassifications was higher for Wiki-Gendersort (82%) than for Gender API (40%) and NamSor (20%). Finally, Gender API and NamSor tended to misclassify more female names than male names, while the reverse was true for Wiki-Gendersort. Removing all unisex names that we were able to identify, the overall performance was slightly higher for NamSor (errorCoded 43% for File #2 versus 53% for File #1), similar for Gender API (66% versus 65%), and slightly lower for Wiki-Gendersort (94% versus 90%).

Due to a large number of nonclassifications, we repeated the analyses with only the second Chinese character translated into Pinyin for two-character given names whose gender could not be determined. Appendix 1 shows the confusion matrices and Appendix 2 shows the performance metrics. With this approach, the number of nonclassifications was reduced for all three tools (from 7,951 to 70 for Gender API, from 4,001 to 0 for NamSor, and from 16,342 to 6,543 for Wiki-Gendersort). Yet, in return, the number of misclassifications increased (from 5,040 to 8,048 for Gender API, from 6,504 to 8,143 for NamSor, and from 1,712 to 5,517 for Wiki-Gendersort). Overall, the performance of the three tools, as estimated by errorCoded, improved due to the marked decrease in the number of nonclassifications. The proportion of errors decreased from 65% to 41% for Gender API, from 53% to 41% for NamSor, and from 90% to 60% for Wiki-Gendersort.

## DISCUSSION

### Main findings

We found that all three tools tested in this study inaccurately predicted the gender of Chinese given names. The overall proportion of errors (misclassifications and nonclassifications) was 53% for NamSor, 65% for Gender API, and 90% for Wiki-Gendersort. There were no substantial differences in results when using a data file with or without the unisex names we were able to identify. Repeating the analyses with only the second Chinese character translated into Pinyin for nonclassifications yielded better results, as the overall proportion of errors was reduced to 41% for NamSor and Gender API and to 60% for Wiki-Gendersort. Misclassifications were common for both male and female names, although Gender API and especially NamSor tended to make more errors with female names and Wiki-Gendersort with male names.

### Comparison with existing literature

To our knowledge, there are little data in the literature regarding the accuracy of gender detection tools, and even less data for non-Western given names. As our previous study included only 7% Asian and 5% Arabic names, we refrained from calculating performance metrics for these names [7]. In a recent paper, Santamaría and Mihaljević compared the performance of five gender detection tools (NamSor, Gender API, genderize.io, gender-guesser, and NameAPI) using a database of 7,076 given names, of which 2,304 (34%) were considered Asian [6]. The authors used NamSor's origin API to determine the origin of the names and found that performance was significantly lower for Asian names compared with European names. For Gender API, the best performing tool in their study, errorCoded was only 2.8% for European names but 17.6% for Asian names. For NamSor, the second best performing tool, these values were 2.7% and 34.6%, respectively.

Our results showed even poorer performance (Gender API: 65.0% in our study versus 17.6% in Santamaría and Mihaljević's study, NamSor: 52.5% versus 34.6%). These differences may be partly explained by errors in the categorization of names in Santamaría and Mihaljević's study (for example, non-Asian names incorrectly considered as Asian by NamSor's origin API) or differences in performance according to the Asian countries considered. Our study focused only on Chinese names, which was not the case in Santamaría and Mihaljević's study.

### Implications for practice

The three gender detection tools evaluated in this study, which performed well with Western given names as demonstrated by Santamaría and Mihaljević's and our own study [6, 7], inaccurately determined the gender of Chinese given names and therefore should not be used in this population. The procedure of using only the second Chinese character translated into Pinyin for nonclassifications led to better results, but the proportion of errors was still far too high to recommend the use of these tools for Chinese given names.

Two gender detection tools (Gender API and NamSor) use additional parameters regarding the accuracy of the estimate, whereas Wiki-Gendersort does not. We believe that these parameters are not useful for determining the gender of Chinese given names, for three reasons. To our knowledge, there are no threshold values above which the estimate can be considered “sufficiently accurate.” In addition, the use of threshold values would move a large proportion of classified given names into the nonclassification group, even though the level of inaccuracy, as found in our study, was already high for all three tools. Finally, the mean level of accuracy estimated by these parameters was only slightly higher in the correct classification group (“accuracy” 83% for Gender API and “probabilityCalibrated” 78% for NamSor) compared with the misclassification group (81% and 76%, respectively). These accuracy parameters are therefore of little use in separating correctly classified names from misclassified names.

As our study focused on Chinese names, the results cannot be transposed to other non-Western countries. Therefore, it would be useful to evaluate the performance of gender detection tools with names of people from other countries that are prominent in science and engineering, such as Japan, South Korea, or India.

### Limitations

The study has some limitations that should be mentioned. The study relied on a gender-labeled database of 172,624 Chinese given names compiled from several public sources in China. Pypinyin was used to translate the Chinese names into Pinyin format. The use of this database could be a source of errors, especially in the gender assigned to the names in Chinese characters and in the translation of Chinese characters into Pinyin names. Though possible, these errors are probably few. Indeed, Pypinyin is a widely used tool for translating Chinese characters into Pinyin, and two bilingual Chinese/English research assistants found no Pinyin translation or gender assignment errors in a sample of 250 randomly drawn given names (Appendix 3). According to the research assistants, half of the names checked (n=124) were not gender specific. The large number of unisex names in China and the loss of information due to romanization probably account for much of the inaccuracy of gender detection tools with Chinese given names.

Gender detection tools assign a sex (i.e., female or male) to individuals based primarily on their first name and sometimes also on their last name or possible cultural origin. By doing so, these tools oversimplify a much more complex concept (gender). Sex and gender are not, however, interchangeable [21, 22]. Sex is determined by biological aspects of a person, while gender is generally related to sociocultural roles. Therefore, to adopt a more respectful approach, it would be preferable to obtain gender data by self-identification. Unfortunately, self-identification is resource intensive and is not feasible for large-scale studies.

## CONCLUSION

We found that all three gender detection tools evaluated in this study inaccurately predicted the gender of Chinese given names in Pinyin format and therefore should not be used in this population. The overall proportion of errors (errorCoded) was 53% for NamSor, 65% for Gender API, and 90% for Wiki-Gendersort.

## Data Availability

Data associated with this article are available in the Open Science Framework: https://doi.org/10.17605/OSF.IO/ZQVW5.

## SUPPLEMENTAL FILES

**Appendix 1.** Confusion matrices for gender detection tools

**Appendix 2.** Performance metrics for gender detection tools

**Appendix 3.** Pinyin translations and gender assignments in a sample of randomly drawn names
