# Erratum to “How accurate are gender detection tools in predicting the gender for Chinese names? A study with 20,000 given names in Pinyin format,” 2022;110(2):205–11.

**DOI:** 10.5195/jmla.2022.1544

**Published:** 2022-04-01

**Authors:** Charlene Dundek

The PDF version of this article reflected an older version of the article with an incorrect URL for reference 17, while the HTML version was correct. The PDF has been updated to the correct version.

