# Supplementary material for: How accurate are gender detection tools in predicting the gender for Chinese names? A study with 20,000 given names in Pinyin format
Source: J Med Libr Assoc. 2022 Apr 1;110(2):205–11. doi: 10.5195/jmla.2022.1289 (PMC9014919; doi:10.5195/jmla.2022.1289)
Supplement: Supplementary file 1 — Appendix 1. Confusion matrices for gender detection tools [file jmla-110-2-205-s01.pdf]

Appendix 1. Confusion matrices for gender detection tools (n=20,000 given names with only the second Chinese character if the gender could not be determined with the full two-character given name)

Gender detection tool	Classified as women<br>n (%)	Classified as men<br>n (%)	Not classified<br>n (%)
Gender API			
Women	2947 (36.6)	5074 (63.1)	23 (0.3)
Men	2974 (24.9)	8935 (74.7)	47 (0.4)
NamSor			
Women	2278 (28.3)	5766 (71.7)	0
Men	2377 (19.9)	9579 (80.1)	0
Wiki-Gendersort			
Women	951 (11.8)	4302 (53.5)	2791 (34.7)
Men	1215 (10.1)	6989 (58.5)	3752 (31.4)
