# Supplementary material for: How accurate are gender detection tools in predicting the gender for Chinese names? A study with 20,000 given names in Pinyin format
Source: J Med Libr Assoc. 2022 Apr 1;110(2):205–11. doi: 10.5195/jmla.2022.1289 (PMC9014919; doi:10.5195/jmla.2022.1289)
Supplement: Supplementary file 2 — Appendix 2. Performance metrics for gender detection tools [file jmla-110-2-205-s02.pdf]

Appendix 2. Performance metrics for gender detection tools (n=20,000 given names with only the second Chinese character if the gender could not be determined with the full two-character given name)

Gender detection tool	errorCoded	errorCodedWithoutNA	naCoded	errorGenderBias
Gender API	0.4059	0.4038	0.0035	-0.1054
NamSor	0.4072	0.4072	0	-0.1695
Wiki-Gendersort	0.6030	0.4100	0.3272	-0.2294
